# Detection of Group B Streptococcus Directly from Collected ESwab Samples by Use of the BD Max GBS Assay

**DOI:** 10.1128/JCM.00445-16

**Published:** 2016-05-23

**Authors:** Suzane Silbert, Talita T. Rocchetti, Alicia Gostnell, Carly Kubasek, Raymond Widen

**Affiliations:** a Esoteric Testing Laboratory, Pathology Department, Tampa General Hospital, Tampa, Florida, USA; b Laboratório Especial de Microbiologia Clínica (LEMC), Federal University of São Paulo/UNIFESP, São Paulo, Brazil; Boston Children's Hospital

## Abstract

Group B Streptococcus detection directly from Copan ESwab collected samples, using the BD Max GBS assay, was evaluated on receipt in the laboratory and after 24 h at room temperature. Results were compared to those using Lim broth enrichment PCR and culture. No significant difference was observed between 24 h ESwab and Lim broth PCRs.

## TEXT

Group B Streptococcus (GBS) can cause severe disease in a newborn and is known to be a leading cause of life-threatening neonatal bacterial infections. Transient colonization of the female urogenital tract by GBS is a recognized risk factor for the development of infection acquired during the birthing process (1–4). The current standard of care recommended by the Centers for Disease Control and Prevention (CDC) for preventing neonatal GBS disease is screening pregnant women at 35 to 37 weeks of gestation to determine their GBS colonization status (5). Recommended screening consists of swabbing the woman's lower vagina and rectum and incubating the swab in enrichment broth for ≥18 h at 35 to 37°C prior to GBS testing. Available GBS testing includes culture, with or without the aid of a chromogenic medium, and nucleic acid amplification tests (NAATs). Following enrichment, culture-based methods can take from 48 to 72 h for a final result. Molecular-based tests, however, are usually faster than culture (6–12). The BD Max GBS assay (BD Diagnostic Systems, Québec, Canada) performed on the BD Max system (BD Diagnostic Systems, Sparks, MD) is a PCR test intended for use with enriched Lim broth culture after ≥18 h of incubation of vaginal/rectal swab samples and can provide results for 24 specimens in approximately 2.5 h (10, 11, 13).

Advancements in technology in clinical microbiology and molecular laboratories have made available more efficient methods of specimen collection and processing, which along with the detection methods have the potential to improve the sensitivity of screening and the recovery of GBS and other pathogens. The combination of a liquid-based transport medium and flocked swab, such as the ESwab (Copan Diagnostics, Murrieta, CA) collection device, has been demonstrated to increase the recovery of bacteria from clinical sites compared to that of standard fiber-wrapped swabs (14–17). The use of ESwab samples with the BD Max GBS assay may potentially improve and speed up the detection of GBS in pregnant women by eliminating the Lim broth inoculation and incubation steps. The objective of this study was to evaluate the detection of GBS directly from collected ESwab samples, using the BD Max GBS assay, after the following two different time points: (i) same day, i.e., when the sample arrives in the laboratory and (ii) 24 h after ESwab is held for 18 to 24 h at room temperature. The second time was selected to simulate delays associated with the transport of clinical specimens from remote collections sites, such as physician offices. The performance of the BD Max GBS assay using ESwab samples has not been established, and therefore, use of ESwab is outside of the product claims for the BD Max GBS assay.

A total of 410 vaginal and rectal collected ESwab samples that were submitted for GBS testing at the Esoteric Testing Laboratory from Tampa General Hospital (Tampa, FL) during a 7-month period (January to August 2015) were included in this study. Detection of GBS was performed using the BD Max GBS assay following three different procedures: (i) the standard of care Lim broth procedure, (ii) a same-day ESwab direct PCR procedure, and (iii) a 24-h ESwab direct PCR procedure.

In the laboratory, ESwabs were vortexed with transport medium (modified Amies liquid). For the standard-of-care PCR procedure, an aliquot of 200 μl of the ESwab transport medium was inoculated into Lim broth (BD Diagnostic Systems, Sparks, MD) and incubated for ≥18 h at 37°C. Following enrichment, on the second day, a 15-μl aliquot of the Lim broth was mixed with the BD Max GBS sample preparation reagent and processed on the BD Max system using the BD Max GBS assay according to manufacturer's recommendations (13). In addition to PCR, culture was also performed on the second day. An aliquot of 100 μl of the Lim broth was subcultured onto BBL Trypticase soy agar with 5% sheep blood (TSA II) and BBL Columbia CNA agar with 5% sheep blood (BD Diagnostic Systems, Sparks, MD) and was incubated for 48 h at 35 to 37°C. Colonies resembling GBS were identified using Gram stain, catalase, and the BD BBL Streptocard acid latex test (BD Diagnostic Systems). The ideal volume of ESwab medium inoculated in Lim broth was previously validated in our laboratory. Of several dilutions of ESwab with GBS inoculated into Lim broth medium, 200 μl was selected as the optimal volume and is currently used as our standard-of-care procedure for Lim broth culture.

For the second procedure, the same-day ESwab direct PCR, a 400-μl aliquot of the ESwab medium was inoculated directly into the BD Max GBS sample preparation reagent, skipping the enrichment period. The sample was processed on the BD Max system using the BD Max GBS assay. Sample collection time and same-day direct PCR run time were recorded for all samples included in this study, and an average of time between sample collections and same day direct PCR runs was calculated. The third procedure, which was the 24-h ESwab direct PCR, was performed with the remaining ESwab medium after ESwab was held for 24 h at room temperature. For this test, a 200-μl aliquot of the 24-h ESwab medium was added to the BD Max GBS sample preparation reagent and processed on the BD Max system using the BD Max GBS assay.

Analytical studies were initially done to determine ideal ESwab medium volumes for PCR testing, without compromising the standard-of-care procedure for Lim broth culture. Serial 10-fold dilutions of GBS in ESwab medium were tested fresh and after incubation at room temperature for 24 h. Volumes of 400 μl and 200 μl were determined to be optimal for same-day and 24-h testing, respectively. The findings at 24 h were consistent with prior assumptions that low-level multiplication of the organisms occur at room temperature after receipt in the laboratory.

The sensitivity and specificity of Lim broth PCR and direct ESwab PCR (same day and 24 h) compared to those of culture were determined, and differences were calculated by the McNemar test at a significance level of 0.05 (Table 1). The differences in sensitivity and specificity between 24-h direct ESwab PCR and Lim broth PCR as well as in specificity between same-day direct ESwab PCR and Lim broth PCR were not statistically significant. However, the difference in sensitivity between same-day direct ESwab PCR and Lim broth PCR was statistically significant (*P* = 0.0156), even though same-day direct ESwab PCR had very good sensitivity (92.7%). Standard-of-care PCR, culture, and direct ESwab PCR (same day and 24 h) displayed an overall agreement of 95.6% (*n* = 389). Of these, 288 samples were negative, and 101 samples were positive for GBS by all 3 methods. Discrepant results were observed for 21 samples (Table 2). The average time between sample collection and same-day direct ESwab PCR testing was 7.1 h (range of 0.2 h to 24 h).

**TABLE 1:** Sensitivity and Specificity of direct ESwab PCR and Lim broth PCR with the BD Max GBS assay^*a*^

Comparison	ESwab PCR, %		Lim broth PCR, %		ESwab PCR vs Lim broth PCR, %		McNemar test
	Estimate	95% CI^*b*^	Estimate	95% CI	Difference	95% CI	
Sensitivity^*c*^							
Same-day ESwab vs Lim broth PCR	92.7 (101/109)	86.2–96.2	99.1 (108/109)	95.0–99.8	−6.4	−12.8 to −1.4	0.0156
24-h ESwab vs Lim broth PCR	97.2 (106/109)	92.2–99.1	99.1 (108/109)	95.0–99.8	−1.8	−6.7 to 2.2	0.5000
Specificity^*d*^							
Same-day ESwab vs Lim broth PCR	96.7 (291/301)	94.0–98.2	95.0 (286/301)	91.9–97.0	1.7	−0.1 to 3.8	0.0625
24-h ESwab vs Lim broth PCR	96.0 (289/301)	93.2–97.7	95.0 (286/301)	91.9–97.0	1.0	−0.8 to 3.1	0.375

a Lim broth culture is the reference method.

b 95% CI, 95% confidence interval.

c Values in parentheses represent the ratio of positive results to true positives.

d Values in parentheses represent the ratio of negative results to true negatives.

**TABLE 2:**
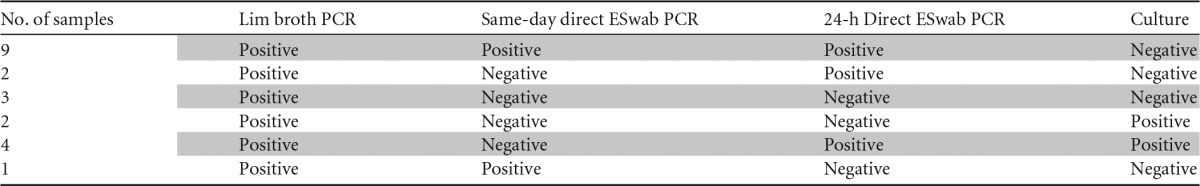
Discrepant results observed among Lim broth PCR, same-day direct ESwab PCR, 24-h direct ESwab PCR, and culture

The implementation of universal screening of pregnant women at 35 to 37 weeks of gestation as recommended by the CDC has significantly reduced the risk of early onset GBS infection in neonates (1–3, 5,). However, some data suggest that there may be missed opportunities for intrapartum antimicrobial prophylaxis (18–20). Several studies have been performed to identify the cause of the remaining infections. A major contributing factor is the failure to seek prenatal care, including routine GBS screening, which affects up to 15% of pregnant women (8–12). In these instances, use of a rapid method for the detection of GBS at the time of delivery may help determine if the administration of prophylaxis during delivery is warranted.

In recent years, a number of FDA-cleared NAATs for GBS detection have been commercialized. An advantage of such tests is that results are available at least 1 day earlier than traditional cultures. In addition, several studies have shown improved GBS detection rates using NAATs compared to those when using culture (6, 10–12, 20). Still, most of the currently available NAATs specify that testing is to be performed on 18- to 24-h enrichment broth cultures as recommended by the CDC. While broth enrichment for 18 to 24 h increases the sensitivity of GBS screening by culture- and molecular-based tests, it also introduces an increase in the turnaround time (TAT), which leaves a gap in care for women with incomplete prenatal care or premature labor (11).

A few studies evaluated direct GBS molecular testing in order to reduce the TAT and to sustain cases of incomplete prenatal care or premature labor (11, 12, 17, 21). An interesting study (20) assessed the accuracy of a direct molecular test by comparing intrapartum specimens tested by the Xpert GBS assay (Cepheid, Sunnyvale, CA) at the onset of labor to the current antenatal culture screening performed with specimens collected at 35 to 37 weeks of gestation. The molecular test presented a sensitivity of 98.5%, while the antenatal culture presented a sensitivity of 58.3% compared to that of intrapartum culture. These results revealed the high sensitivity of a direct molecular test and also described a concern of the antenatal screening. That is, GBS colonization can be intermittent during pregnancy, resulting in a low correlation between antenatal screening results and intrapartum maternal GBS colonization. Similar results were also identified in a different study (22), where the intrapartum molecular test was superior to antenatal cultures (sensitivity of 94% versus 54%). Therefore, it is clear that a rapid intrapartum test is needed. However, the sensitivities of these tests are still controversial (20–22). As opposed to the studies described above, a recent study (12) using the Xpert GBS LB assay (Cepheid) showed that the sensitivity of Lim broth-enriched PCR (99%) was superior to that of direct swab PCR (85.7%).

Buchan et al. (17) tested in parallel vaginal/rectal swabs collected by ESwab and liquid Stuart rayon swabs. Each device was used to directly inoculate culture medium and Lim Broth. ESwab medium inoculation was conducted using an automated system while liquid Stuart was inoculated manually. GBS cultures following Lim broth enrichment were more sensitive than direct inoculation cultures. ESwab collected samples, however, presented greater GBS recovery than liquid Stuart in both, direct, and enriched cultures.

Several studies have demonstrated the superior absorption and release capacities of the Copan ESwab compared to those of traditional swabs (14, 15, 17). ESwabs have shown better recovery and better release of the organism collected. In addition, its transport system also contains liquid medium that is usually compatible with bacterial culture and molecular diagnostic assays. This study considered that the combination of ESwab and BD Max GBS assay could improve and speed up the detection of GBS in pregnant women by skipping the Lim broth inoculation and the ≥18-h incubation steps. We included a second testing direct from ESwab after 24 h held at room temperature to determine if this step would enhance GBS detection if the same-day test was not sufficiently sensitive. It addition, this evaluation would also be useful for specimens shipped to the laboratory from remote sites.

The obtained results suggest that the ESwab system may be a suitable sample collection device alternative for GBS screening in a “sample-in” “answer-out” walk-away real-time PCR assay. Overall, the performances of the same-day and 24-h ESwab direct PCRs were excellent for the detection of GBS using the BD Max GBS assay on the BD Max system. Even though same-day direct testing from ESwab demonstrated a reduce sensitivity (92.7%) compared to those of the 24-h ESwab direct PCR (97.2%) and Lim broth PCR (99.1%), it may be an option for emergency cases or for rapid screening of women at the time of delivery. The ability to test a direct specimen may aid in guiding the administration of prophylaxis in patients who have not been screened prior to delivery and in cases of unexpected or early labor where enrichment culture or PCR results are not yet available. We propose that if the results of the direct test are negative, the test may potentially be repeated after ESwab is held for 24 h at room temperature to confirm direct testing. Alternatively, one may default to the inoculation of Lim broth for 18 to 24 h of incubation prior to PCR testing, which is the recommended standard of care.
